# Research on the Grinding Quality Evaluation of Composite Materials Based on Multi-Scale Texture Fusion Analysis

**DOI:** 10.3390/ma18153540

**Published:** 2025-07-28

**Authors:** Yangjun Wang, Zilu Liu, Li Ling, Anru Guo, Jiacheng Li, Jiachang Liu, Chunju Wang, Mingqiang Pan, Wei Song

**Affiliations:** 1 School of Mechanical and Electrical Engineering, Soochow University, Suzhou 215000, China; wangyangjun@suda.edu.cn (Y.W.); pmqwl@suda.edu.cn (M.P.); sw154609@163.com (W.S.); 2 Institute of Aerospace Materials and Technology, Beijing 100076, China; liuzilu1988@163.com (Z.L.); lingli_fly@163.com (L.L.); 200521025@163.com (A.G.)

**Keywords:** machine vision, composite materials, surface finishing quality, multi-scale texture analysis

## Abstract

To address the challenges of manual inspection dependency, low efficiency, and high costs in evaluating the surface grinding quality of composite materials, this study investigated machine vision-based surface recognition algorithms. We proposed a multi-scale texture fusion analysis algorithm that innovatively integrated luminance analysis with multi-scale texture features through decision-level fusion. Specifically, a modified Rayleigh parameter was developed during luminance analysis to rapidly pre-segment unpolished areas by characterizing surface reflection properties. Furthermore, we enhanced the traditional Otsu algorithm by incorporating global grayscale mean (μ) and standard deviation (σ), overcoming its inherent limitations of exclusive reliance on grayscale histograms and lack of multimodal feature integration. This optimization enables simultaneous detection of specular reflection defects and texture uniformity variations. To improve detection window adaptability across heterogeneous surface regions, we designed a multi-scale texture analysis framework operating at multiple resolutions. Through decision-level fusion of luminance analysis and multi-scale texture evaluation, the proposed algorithm achieved 96% recognition accuracy with >95% reliability, demonstrating robust performance for automated surface grinding quality assessment of composite materials.

## 1. Introduction

The growing demand for lightweight, high-performance spacecraft has established fiber-reinforced composites and honeycomb sandwich structures as core materials for primary load-bearing components, owing to their exceptional specific strength and corrosion resistance [1,2,3]. However, the reliability and service life of these structures critically depend on the bonding quality between composite components. Surface grinding is widely employed to enhance interfacial bonding strength and fatigue life by removing contaminants, controlling roughness, and generating microscopic mechanical interlocking structures [4]. Studies confirm that adhesive performance is significantly influenced by the uniformity of surface topography and multi-scale textural characteristics. Conversely, excessive abrasion or uneven texture distribution may induce stress concentration [5], potentially leading to premature interfacial failure. Current surface grinding quality assessment predominantly relies on contact profilometers or empirical visual inspection—methods limited by low efficiency, localized sampling bias, and inadequate characterization of full-field textural features [6]. Particularly for complex-curvature spacecraft components, conventional methods fail to enable non-contact, high-precision, real-time in situ inspection [7]. In recent years, machine vision-based nondestructive evaluation techniques have gained prominence in surface quality assessment due to their high-resolution imaging, full-field analysis capabilities, and superior morphological feature extraction [8,9]. However, significant challenges persist in characterizing complex textures, integrating multi-scale feature integration, and morphological quantification. For instance, abraded surfaces contain multi-scale textures ranging from micrometer-scale grooves to millimeter-scale undulations, where single-scale analysis tends to overlook critical features [10]. Additionally, illumination conditions, material anisotropy, and surface reflectivity may distort image intensity [11], adversely affecting texture feature extraction accuracy.

In this study, the composite components exhibit surface reflectivity with directional scattering characteristics. Therefore, we proposed a luminance analysis method incorporating a modified Rayleigh distribution, which enables rapid pre-segmentation of non-abraded surfaces under experimental illumination conditions. Subsequently, the conventional Otsu algorithm for global threshold segmentation based on grayscale histograms has been enhanced by integrating texture and gradient modal features. Through multi-scale texture analysis, the hierarchical structural information of abraded surfaces is systematically captured. Unlike traditional evaluation algorithms based on the correlation between image features and surface roughness, this study innovatively incorporates luminance and multi-scale texture fusion analysis, significantly enhancing the recognition accuracy for different features of material surfaces.

## 2. Algorithm Core Framework

**Figure 1:**
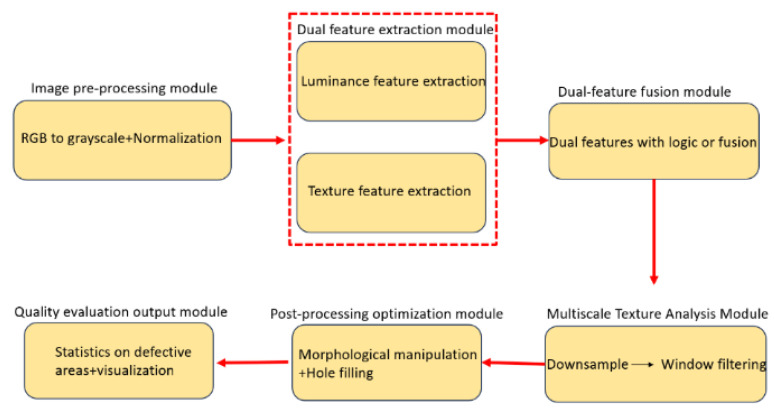
Core structure of algorithms.

Conventional image processing methodologies primarily encompass template matching, edge detection, and texture analysis. For instance, template matching evaluates quality by comparing deviations between reference templates and target images, while texture analysis quantifies surface roughness and uniformity [12]. However, these approaches demonstrate constrained generalization capabilities due to their reliance on manual feature engineering. Statistics-based grayscale histogram analysis reflects surface finish, and edge histogram methods assess edge distribution uniformity, yet such techniques prove inadequate for processing images under high dynamic range (HDR) scenarios or complex illumination conditions [13]. This paper develops a surface polishing quality evaluation algorithm grounded in multi-scale texture fusion, which strategically adapts to varying material dimensions through differentiated assessment protocols. Capitalizing on the discriminative advantages of texture analysis, the framework dynamically selects evaluation metrics based on workpiece scale: direct root mean square error (RMSE) quantification governs local texture variation characterization for smaller specimens, while an integrated luminance–texture fusion decision architecture orchestrates quality assessment for larger-scale materials. The operational paradigm of this scale-adaptive algorithm is systematically depicted in Figure 1, demonstrating the coherent integration of multi-modal texture features through cascaded fusion modules.

### 2.1. Texture Feature Analysis

For fiber-reinforced polymer (FRP) composites with smaller dimensions where surface luminance exhibits negligible impact on subsequent image processing, localized root mean square error (RMSE) quantification can be adopted as the principal textural metric to characterize regional texture variations. The FRP material, composed of a polymer matrix reinforced with glass fibers, demonstrates three predominant surface quality defects during polishing processes: (1) directional striated textures caused by exposed fiber bundles, (2) cloud-like mottling from resin inhomogeneity, and (3) linear scratch patterns induced by mechanical abrasion. Whereas conventional grayscale statistical parameters prove inadequate in discriminating these defect modalities, the RMSE feature demonstrates enhanced discriminative capacity through systematic quantification of localized intensity fluctuations, thereby facilitating composite texture pattern characterization [2].

(1) $$RMSE\left(x,y\right)=\sqrt{\frac{1}{N}\Sigma_{i=1}^{N}{\left(y_{i}-{\hat{y}}_{i}\right)}^{2}}$$

where N represents the number of data points, $y_{i}$ denotes the i actual measurement value, and ${\hat{y}}_{i}$ corresponds to the i predicted or expected value. In fiber-exposed regions of material surfaces, localized high-frequency directional grayscale transitions (caused by fiber bundle protrusions) induce significantly elevated RMSE values, whereas resin-rich areas exhibit low-frequency grayscale gradients due to residual matrix accumulation. This differential response enables preliminary quality assessment through spatial RMSE distribution analysis.

### 2.2. Luminance Feature Characterization

The composite materials investigated in this study exhibit distinct optical behaviors depending on their surface conditions. Non-abraded surfaces approximate specular optical surfaces, producing concentrated directional reflectance that manifests as high-luminance regions during imaging. In contrast, abraded surfaces predominantly demonstrate diffuse reflection due to microscale structural modifications [14]. For fiber-reinforced polymers (FRPs), surface abrasion generates microstructural features—including fiber alignment variations and resin–fiber interface irregularities—which induce stochastic light scattering distributions.

The scale parameter σ in the Rayleigh distribution directly correlates with surface roughness metrics. Elevated σ values signify heightened scattering intensity, characteristic of non-abraded regions with high specular reflectance. Conversely, diminished σ values correspond to the uniform diffuse reflectance observed on abraded surfaces, enabling quantitative discrimination of light-scattering behaviors across progressive abrasion stages. To address directional scattering patterns inherent in composite surfaces, a modified Rayleigh distribution is introduced into the luminance analysis framework. This adaptation enhances the precision of reflectance characterization, particularly for surfaces with anisotropic scattering properties. The probability density function of the Rayleigh distribution is defined as follows [15]:

(2) $$p\left(I\right)=\frac{2I}{\sigma^{2}}e^{-\frac{I^{2}}{\sigma^{2}}},I\geq 0$$

The scale parameter σ, central to the Rayleigh distribution, is derived from the standard deviations (denoted as X and Y) of two independent orthogonal Gaussian variables. In this experimental framework, σ serves as a surface roughness proxy parameter. Post-abrasion surface modifications induce an increase in σ values, which correspond to diminished high-luminance regions caused by reduced specular reflectance. This parametric relationship enables rapid localization of specular reflection zones through adaptive luminance threshold segmentation.

### 2.3. Enhanced Dual-Threshold Otsu Algorithm

The Otsu algorithm, fundamentally based on grayscale histogram analysis, operates by maximizing inter-class variance to optimally separate foreground features (e.g., non-abraded specular regions) from background materials. However, conventional implementations are constrained by single-modal sensitivity—relying exclusively on grayscale information without incorporating complementary texture and gradient features. This limitation becomes particularly critical in composite material analysis, where localized specular highlights and substrate textures may exhibit analogous grayscale values, inducing misclassification. Furthermore, surface abrasion-induced micro-scratches and noise artifacts distort grayscale distributions, frequently generating multimodal histogram profiles that challenge traditional Otsu’s unimodal threshold selection, as evidenced by experimental specimens with manually abraded surfaces exhibiting heterogeneous abrasion levels:

(3) $$T_{Pre}=\mu +2\sigma$$

Within the pre-segmented region satisfying I≥Tprⅇ, where μ denotes the global grayscale mean and σ represents the grayscale standard deviation, the optimal secondary threshold $T_{b}$ is determined through constrained Otsu optimization [12]:

(4) $$T_{b}=\mathrm{argmax}\left\{\omega_{l}\left(T\right)\omega_{h}\left(T\right){\left[\mu_{l}\left(T\right)-\mu_{h}\left(T\right)\right]}^{2}\right\}$$

Let $\omega_{l}$ and $\omega_{h\;}$ denote the pixel proportion weights of low- and high-luminance regions, respectively, with $\mu_{l}$ and $\mu_{h}$ representing their intra-class mean intensities. The enhanced algorithm achieves rapid pre-segmentation of high-luminance zones, followed by synergistic fusion of luminance signatures and multi-scale texture descriptors through logical OR operations, enabling concurrent detection of specular anomalies and textural. A comparison between the traditional Otsu algorithm and the improved Otsu algorithm proposed in this study is shown in Table 1.

### 2.4. Multi-Scale Texture Characterization

Multi-scale texture analysis is a technique that extracts textural features across varying spatial resolutions or frequency domains, enabling comprehensive characterization of hierarchical surface structures from macroscopic to microscopic levels. By integrating texture features from different scales, this approach achieves a more complete representation of complex surfaces [14]. In this study, unabraded composite specimens exhibit physical pit defects with significant dimensional variations (0.1–2 mm), which appear as discrete points in imaging data. Under high-resolution (microscale) observation, these features display sharp edges but demonstrate noise sensitivity, whereas lower-resolution (macroscale) analysis yields blurred textures with enhanced noise immunity. Conventional single-scale windowing methods fail to concurrently capture multi-dimensional features: smaller windows exhibit suboptimal window sizing effects—missing large-scale textures while amplifying noise interference—whereas larger windows cause detail loss and positioning inaccuracy.

The proposed algorithm employs a three-tier Gaussian pyramid structure to construct scale space, generating multi-scale image representations at original resolution, 1/2 down sampling, and 1/4 down sampling levels. Each pyramid tier is processed using the following equation [14]:

(5) $$G_{L}\left(x,y\right)=\sum_{m,n}\omega \left(m,n\right)\cdot G_{L-1}\left(2x+m,2y+n\right)$$

Let $L\epsilon \left\{0,1,2\right\}$ denote the pyramid layer index, where L=0 corresponds to the original resolution image. The scale transition is governed by a 5 × 5 Gaussian kernel $\omega \left(m,n\right)$ with spatial coordinates $\left(m,n\right)\epsilon {\left[-2,2\right]}^{2}$, ensuring scale-space continuity.

Post-abrasion composite surfaces exhibit significant textural complexity gradients, rendering fixed-size windowing ineffective for simultaneously accommodating macroscale characterization and microscale preservation. Unabraded high-reflectance zones necessitate large windows (e.g., 60 × 60 pixels at 1/4 scale) to capture global signatures, while micro-scratches demand smaller windows (15 × 15 pixels at original scale) to retain edge fidelity. To address this, the proposed algorithm implements a scale-adaptive windowing scheme with geometrically progressive dimensions, while maintaining consistent physical dimensions [10]:

(6) $$W_{L}=W_{0}\cdot 2^{-L}\left(1+0.2L\right)$$

Let $W_{0}$ denote the base window size at the original scale, and 0.2L represent the scale compensation factor to mitigate window undersizing at higher pyramid levels. The local texture standard deviation at scale L is calculated as follows [10]:

(7) $$\sigma_{L}\left(x,y\right)=\sqrt{\frac{1}{W_{L}^{2}}\sum_{i,j}{\left[I_{L}\left(x+i,y+j\right)-\mu_{L}\right]}^{2}}$$

The local mean $\mu_{L}$ quantifies the intensity uniformity within the analysis window. The maximum fusion strategy, defined in Equation (7), operates under the principle of saliency preservation [10]:

(8) $$\sigma_{fused}\left(x,y\right)=\max\left\{\sigma_{0}\left(x,y\right),\sigma_{1}\left(x,y\right),\sigma_{2}\left(x,y\right)\right\}$$

This strategy preserves salient features across scales through mathematical equivalence to parallel detector configurations, effectively enhancing defect recall rates.

The algorithm comprehensively assesses composite surface abrasion quality by fusing luminance and texture features through logical OR operation: $M_{final}=M_{bright}\vee M_{texture}$, effectively preventing detection omissions inherent to single-feature methods while ensuring high recall rates. Morphological post-processing guarantees geometric compatibility between structural elements and textural characteristics, enhancing detection accuracy and robustness. Including an opening operation with structural element $B_{\mathrm{open}}=disk(r_{1}),r_{1}=3\;pixels$ to eliminate discrete noise points, and a closing operation $B_{\mathrm{close}}=disk(r_{2}),r_{2}=5\;pixels$. The radius relationship derivation formula is as follows [12]:

(9) $$r_{2}=r_{1}+\frac{median\left(W_{L}\right)}{4}$$

## 3. Experiments

### 3.1. Experimental Image Acquisition

**Figure 2:**
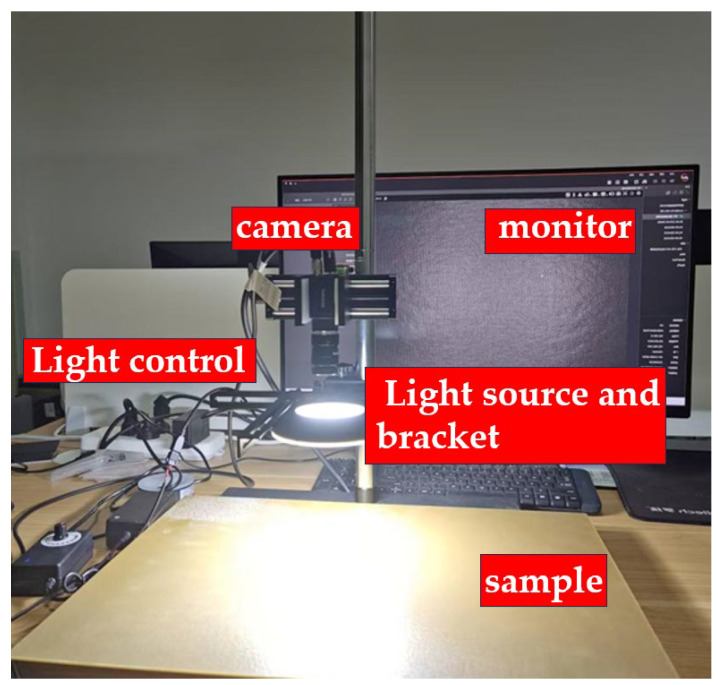
Image acquisition system.

In the present study, all images processed by the algorithm were acquired using the image acquisition system illustrated in Figure 2. This system is primarily composed of a Hikrobot MV-CU200-20GM monochrome camera, a Huakang Technology ring light source (model R120-72-23), and an 8 mm focal length.

C-Mount lens, a mounting bracket, computer hardware (Intel i7-12700H, RTX 3060 Laptop GPU), and a MATLAB-based software development environment that integrates the Image Processing Toolbox along with custom algorithm modules. Detailed parameters of the camera and lens are provided in Table 2. Prior to image acquisition, different areas of the sample were manually polished in an irregular manner to obtain a rich dataset.

**Figure 3:**
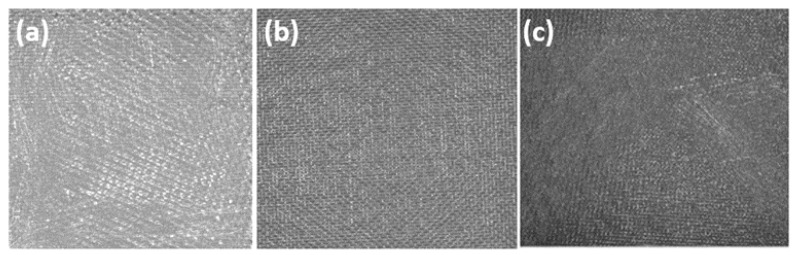
(**a**) Fully sanded texture. (**b**) Completely unsanded texture. (**c**) Mixed and unsanded texture.

The acquisition scheme is as follows: the composite material sample is placed face-up on the bracket support plate. The resin parameters for the glass fiber reinforced polymer (GFRP) composites are presented in Table 3. After adjusting the sample’s position, it is kept stationary while images are manually captured. The sample position is then adjusted, and the process is repeated. For the lighting scheme, the Huakang Technology ring light source R120-72-23 is used, with the camera capturing the light signals reflected from the sample’s surface after being emitted by the light source. The captured images are shown in Figure 3. Moreover, the algorithm developed in this study exhibits strong adaptability to varying lighting conditions.

Due to variations in the applied polishing force on the sample surface, different texture features emerge, which can be categorized into three conditions: unpolished, moderately polished, and over-polished. In this experiment, twenty groups of 2400 × 1400 BMP sample images were acquired, with each group exhibiting distinct visual characteristics—namely, the simultaneous presence of both polished and unpolished texture features, as illustrated in Figure 3c.

### 3.2. Experimental Environment and Evaluation Metrics

In this experiment, the computer hardware configuration is as follows: the CPU is an Intel i7-12700H, and the GPU is an RTX 3060 Laptop. The core software environment is MATLAB R2023b, which integrates the Image Processing Toolbox along with custom algorithm modules to support real-time image analysis and visualization. During the experiment, the parameters for multi-scale ratios were set to 1, 0.5, and 0.25; the window size parameters were configured at 15, 30, and 60; the brightness threshold was set to 0.65 based on environmental variables such as the light source; and the overall standard deviation threshold was set to 0.2. The acquired images were subsequently analyzed and processed, where the algorithm designates polished areas in red and unpolished areas in blue. The accuracy of the algorithm, denoted as P, is evaluated by comparing the red-to-blue area ratio A_1_ with the actual polished-to-unpolished area ratio A_2_, as illustrated in the following formula:

(10) $$P=1-\left|\frac{A_{1}-A_{2}}{A_{2}}\right|$$

In evaluating reliability, the reliability (C) is defined as the ratio of the number of experimental groups whose extracted features match the designed features ($N_{\mathrm{satisfying}}$) to the total number of experimental groups ($N_{all}$), as shown in the following formula:

(11) $$C=\frac{N_{\mathrm{satisfying}}}{N_{all}}$$

### 3.3. Experimental Reliability Analysis

#### 3.3.1. Analysis of RMSE-Based Results

**Figure 4:**
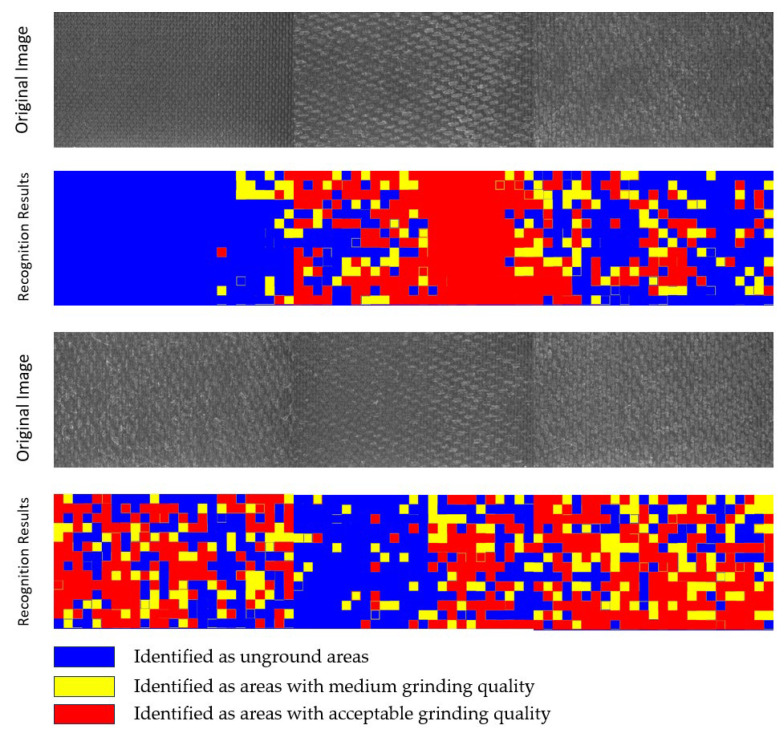
Recognition results.

For fiber-reinforced polymer (FRP) composites with smaller dimensions where luminance interference remains negligible, a multi-scale analytical framework incorporating RMSE-based image processing enables effective visual evaluation of surface polishing quality. The assessment system utilizes chromatic encoding where red designates fully polished zones, yellow signifies adequately processed regions, and blue marks unpolished or substandard areas, with systematic quantification of incomplete polishing ratios. Furthermore, each evaluated image is segmented into 16 standardized sectors, triggering re-polishing protocols when any sector exceeds a 30% threshold of defective area coverage. Experimental results validate the technical validity and operational feasibility of this surface quality assessment methodology across diverse polishing scenarios, as empirically validated in Figure 4.

#### 3.3.2. Reliability Analysis Based on Dual Feature Fusion

**Figure 5:**
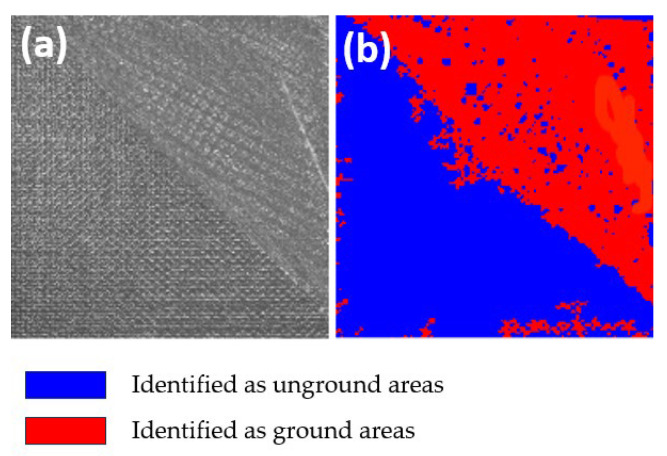
Accuracy test chart. (**a**) Original image. (**b**) Recognition results.

First, the accuracy of the evaluation algorithm was tested. In the test images, certain areas of the surface were fully polished with A_2_ set to 1.0. After feature extraction and computation by the evaluation algorithm, A_1_ was determined to be 0.961, which corresponds to an accuracy of 96.1%. Since this exceeds the predetermined accuracy threshold of 95%, the obtained processing quality characterization parameters are deemed accurate. The experimental results are shown in Figure 5.

**Figure 6:**
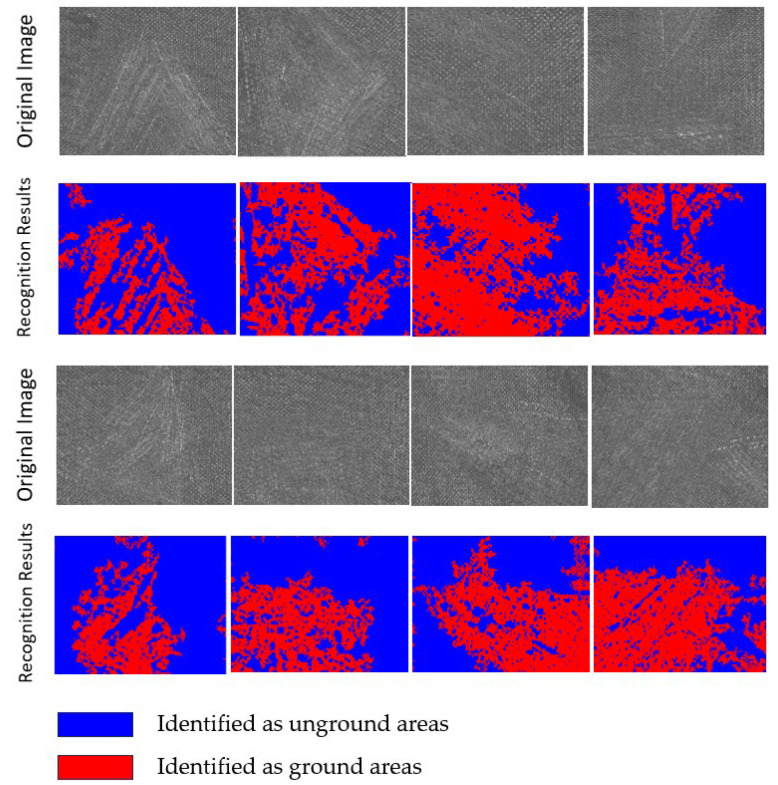
Part of the original image and the recognition result.

Different images were captured from multiple polishing regions, and the evaluation algorithm was applied to extract features from each image. If the extracted features substantially matched the designed features, the corresponding group was counted toward $N_{\mathrm{satisfying}}$. Finally, the reliability of the evaluation algorithm was calculated. Some of the original images and the corresponding recognition results are shown in Figure 6. Comparative analysis of this algorithm versus other algorithms is presented in Table 4.

## 4. Conclusions

Resolving the limitations of traditional methods—such as the low coverage of contact profilometers limited to point sampling (unable to capture full-field texture gradients) and the subjectivity of visual inspection at macroscopic scales with blind spots for micro-defects—our algorithm achieves pixel-level full-field analysis with high resolution, rapid processing, and superior accuracy. Integrated with robotic arms on the production line, this algorithm accomplishes fully automated inspection and grinding operations.

This study utilizes machine vision technology and employs an innovative multi-scale texture fusion analysis method to evaluate the polishing quality of composite material surfaces.

1. Leveraging the optical characteristics of the material surface, the algorithm incorporates brightness analysis combined with a modified Rayleigh distribution to perform preliminary segmentation of unpolished high-brightness regions, thereby improving recognition efficiency.
2. An optimized Otsu algorithm is introduced, incorporating global gray value μ and standard deviation σ. This modification overcomes the traditional Otsu algorithm’s limitations—its reliance on single gray histograms and its ineffectiveness at integrating multimodal features such as texture and gradient—enabling the simultaneous detection of both specular reflection defects and texture uniformity variations.
3. A maximum value fusion strategy integrates features extracted across different scales. This approach effectively preserves significant features at window sizes of 15, 30, and 60, improving the detection rate of minor pits.
4. By optimizing algorithm parameters based on environmental conditions, the experimental accuracy was increased to 96%, while algorithm reliability reached over 95%.

## Figures and Tables

**Table 1:** Comparison between the traditional Otsu algorithm and the enhanced Otsu algorithm.

Limitation Category	Traditional Otsu	Enhanced Otsu Solution
Noise Sensitivity	High noise sensitivity	morphological preprocessing
Uneven Illumination	Single global threshold	Local adaptive thresholding
Multi-threshold Limitation	Binary segmentation only	Watershed post-processing
Material-Specific Adaptation	Fixed threshold failure	Gray histogram redistribution

**Table 2:** Image acquisition camera and lens parameter table.

Property	Value
Resolution	5120 × 3840
Pixel size/μm	1.4
Working distance/mm	185 ± 1
Frame rate/(frame·s^−1^)	5.9
Field of view/mm^2^	107.5 × 80.2
Unit pixel represents physical distance/μm	20.8

**Table 3:** Resin parameters.

Property	Value
Ply Thickness	120 ± 5 μm
Average Resin Layer Thickness	15 ± 3 μm
Fiber Volume Fraction	60 ± 2%

**Table 4:** Comparison of different algorithms.

Algorithm	Accuracy	Reliability	Computational Efficiency
Our Method	96.0%	>95%	185 ms/frame
Otsu Method	82.3%	78%	92 ms/frame
Improved Faster R-CNN	89.7%	85%	320 ms/frame
Swin-MFINet	91.5%	88%	410 ms/frame

## Data Availability

The original contributions presented in this study are included in the article. Further inquiries can be directed to the corresponding authors.
